# A New Green Ionic Liquid-Based Corrosion Inhibitor for Steel in Acidic Environments

**DOI:** 10.3390/molecules200611131

**Published:** 2015-06-17

**Authors:** Ayman M. Atta, Gamal A. El-Mahdy, Hamad A. Al-Lohedan, Abdel Rahman O. Ezzat

**Affiliations:** 1 Surfactants Research Chair, Department of Chemistry, College of Science, King Saud University, P.O. Box 2455, Riyadh 11451, Saudi Arabia; E-Mails: aatta@ksu.edu.sa (A.M.A.); hlohedan@ksu.edu.sa (H.A.A.-L.); ao_ezzat@yahoo.com (A.R.O.E.); 2 Petroleum Application Department, Egyptian Petroleum Research Institute, Cairo 11727, Egypt; 3 Department of Chemistry, Faculty of Science, Helwan University, Helwan 11795, Egypt

**Keywords:** steel, aggregation, contact angle, hydrophobic ionic liquids, potentiodynamic test, EIS

## Abstract

This work examines the use of new hydrophobic ionic liquid derivatives, namely octadecylammonium tosylate (ODA-TS) and oleylammonium tosylate (OA-TS) for corrosion protection of steel in 1 M hydrochloric acid solution. Their chemical structures were determined from NMR analyses. The surface activity characteristics of the prepared ODA-TS and OA-TS were evaluated from conductance, surface tension and contact angle measurements. The data indicate the presence of a double bond in the chemical structure of OA-TS modified its surface activity parameters. Potentiodynamic polarization, electrochemical impedance spectroscopy (EIS) measurements, scanning electron microscope (SEM), Energy dispersive X-rays (EDX) analysis and contact angle measurements were utilized to investigate the corrosion protection performance of ODA-TS and OA-TS on steel in acidic solution. The OA-TS and ODA-TS compounds showed good protection performance in acidic chloride solution due to formation of an inhibitive film on the steel surface.

## 1. Introduction

Carbon steel is a material commonly used for the production and transportation of crude oil in the oil industry and natural gas due to its excellent mechanical properties [1,2,3,4]. Several problems occur during transportation of crude oil in the pipelines, where the migrating ions come into contact with metal due to the breakdown of the oil-aqueous emulsion, which stimulates the corrosion process [5] Furthermore, the corrosion is enhanced by the presence of trace water and salts in the oil and the acidic media which are used in descaling and oil well acidification [6,7]. In a strong acid medium, the corrosion processes produce structural damage to the steel. There are several types of corrosion inhibitors which are widely used to control the corrosion problem of low carbon steel upon exposure to acidic solutions, which vary from organic macromolecules to nanocomposites [8,9,10,11,12,13,14,15]. These compounds are adsorbed onto the metallic surface, blocking the active corrosion sites. The applicability of these materials as corrosion inhibitors for metals in acidic media has been recognized for a long time. However, most of these materials are heavily toxic and environmentally hazardous [16], therefore, attempts have been carried out to search for eco-friendly treatment materials for metals in acid solutions. Recently, ionic liquid (IL)-based products have been developed to solve this problem [17]. ILs are organic salts with negligible vapor pressure that have a melting point below 100 °C, which makes them less hazardous inhibitors and eco-friendlier metal corrosion inhibitors [17]. Moreover, they have a large number of advantageous physicochemical properties such as non-flammability and high ionic conductivity, as well as excellent thermal and chemical stability [18,19,20]. Ionic liquids (ILs) are organic salts containing both organic and inorganic components with various functional groups. Most of these salts are based on imidazolium and pyridinium species as cations, while typical anions are sulfonium, phosphonium, Al_2_Cl_7_, tetrafluoroborate, and bis(trifluoromethane-sulfonyl)imide [17]. ILs are generally considered to be efficient corrosion inhibitors for various metals and alloys due to their high activity in acidic media [21,22,23,24,25]. There are several works reporting the quaternization of alkyl amines with different organic cations to produce quaternary ammonium organic salts like ILs [25,26,27,28,29]. The aim of this work was to prepare ionic liquids to protect steel from corrosion in an acidic environment by reacting octadecyl or oleylamine with *p*-toluenesulfonic acid. To our knowledge no information is available about the corrosion inhibition characteristics of these materials, so electrochemical and surface characteristic techniques were used to investigate the inhibition effects of such materials in 1 M HCl aqueous solution.

## 2. Results and Discussion

### 2.1. Preparation of Octadecylammonium Tosylate (ODA-TS) and Oleylammonium Tosylate (OA-TS)

**Scheme 1:**
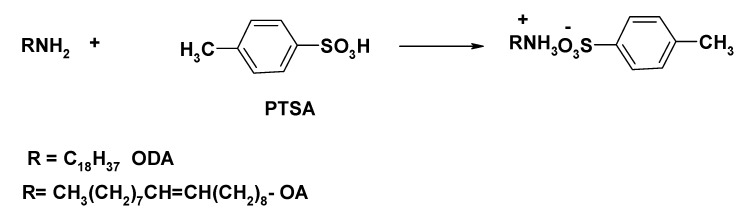
Reaction scheme of OA-TS and ODA-TS.

The preparation of ILs from ODA or OA can be performed by the interaction of an amino group free electron pair with the H^+^ of PTSA with the formation of a new quaternized amine bond. This type of reaction proceeds effectively in the absence of solvent. The reaction scheme was presented in Scheme 1.

**Figure 1:**
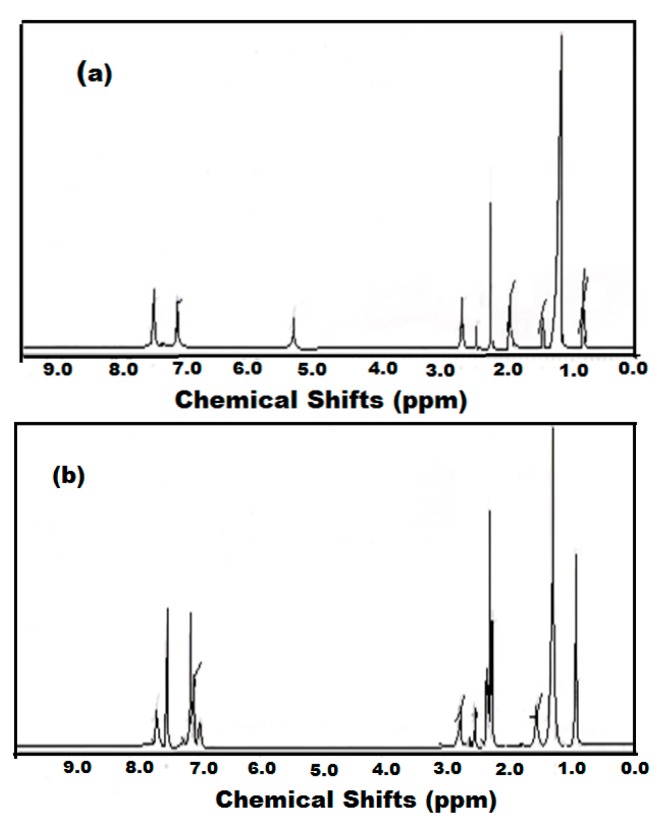
^1^H-NMR spectra of (**a**) OA-TS and (**b**) ODA-TS.

**Figure 2:**
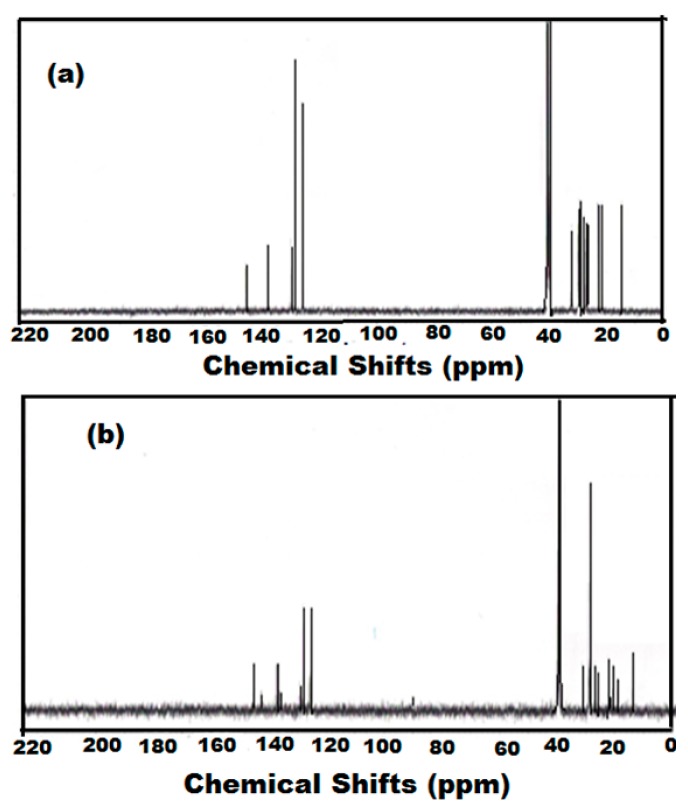
^13^C-NMR spectra of (**a**) OA-TS and (**b**) ODA-TS.

The molecular structures of the compounds were characterized by NMR. The ^1^H-NMR and ^13^C-NMR spectra of ODA-TS and OA-TS are represented in Figure 1 and Figure 2. In the ^1^H-NMR spectra (Figure 1) the peaks appearing at chemical shifts (δ) of 7.709 ppm (doublet, 2H, *J* = 8.1 Hz), and 7.24 ppm (doublet of doublets, 2H, *J* = 8.4, 0.6 Hz, HW) represent the phenyl protons, indicating the incorporation of PTSA as anion in the chemical structures of ODA-TS and OA-TS. Moreover the appearance of peaks at 2.892 (triplet, 2H, *J* = 6.9 Hz) and 2.374 ppm (singlet, 3H) represent the formation of CH_2_-N and ^+^NH_3_ moieties, respectively, confirming the formation of the cation part of ODA-TS and OA-TS. The peaks at 1.628 ppm (multiplet, 2H, *J* = 7.2 Hz), 1.290 ppm (triplet, 30H, *J* = 6.6 Hz), and 0.902 ppm (triplet, 3H, *J* = 6.6 Hz) represent the (CH_2_)_16_ and CH_3_ groups, respectively, confirming the presence of the alkyl chains of ODA and OA without degradation. New peaks at 5.1 ppm in the spectrum of OA-TS (Figure 1a) which represent –CH=CH- protons conform the presence of unoxidized vinyl groups. The ^13^C-NMR spectra (Figure 2) showed the appearance of peaks at δ = 143.63, 141.86, 129.99 and 127.09 ppm which confirm the presence of phenyl and C-N carbons, respectively. The new peak at 132.23 ppm (Figure 2a) confirms the presence of –CH=CH- in the chemical structure of unoxidized OA-TS. The peaks at 40.92, 33.23, 30.95, 30.69, 30.39, 28.72, 27.60, 23.89, 21.49 and 14.61 ppm in the spectra of both OA-TS and ODA-TS indicate the presence of CH_2_ and CH_3_ carbons that are not fragmented during the synthesis procedure.

### 2.2. Surface Properties of OA-TS and ODA-TS

**Figure 3:**
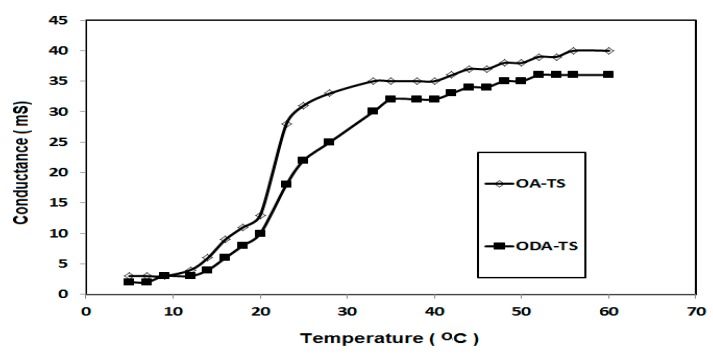
Relation between conductance of ODA-TS and OA-TS and solubility temperature at concentration of 1 mmol·dm^−3^.

The solubility of OA-TS and ODA-TS in water depends on the temperature of the solution. It was observed that at the concentrations of OA-TS and ODA-TS below 1.3 mmol·dm^−3^ they are completely soluble in water at room temperature. However, at above these concentrations the OA-TS and ODA-TS are initially insoluble, and there is often a temperature at which the solubility suddenly increases very dramatically. Accordingly, it is necessary to determine the Krafft temperature, T_K_, at which the solubility of the OA-TS and ODA-TS increased. The T_K_ was determined from conductivity measurements as described in the Experimental section. The relation between conductivity of OA-TS or ODA-TS and the solubility temperature is represented in Figure 3.

**Figure 4:**
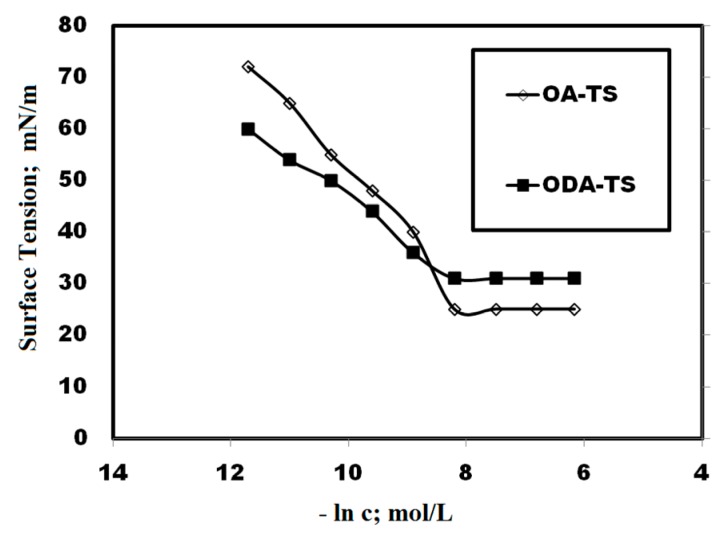
Adsorption isotherms of OA-TS and ODA-TS at temperature of 25 °C.

**Figure 5:**
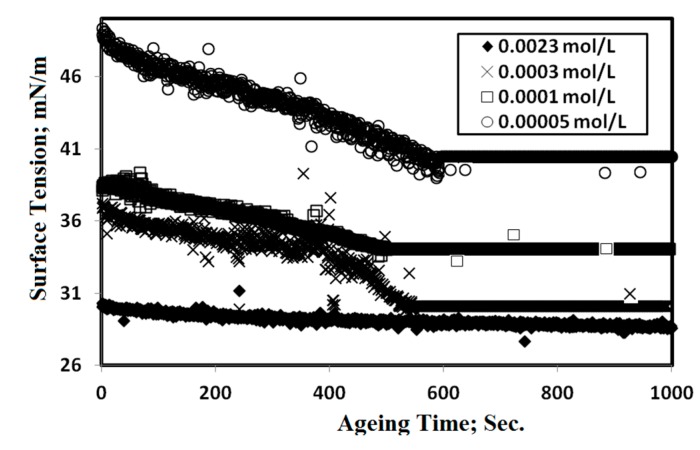
Relation between surface tension and ageing time of different aqueous solutions of OA-TS at 25 °C.

The values of T_K_ for OA-TS and ODA-TS without any salts were 33 and 38 °C, which indicated that OA-TS is more soluble than ODA-TS in water. The solubility of OA-TS and ODA-TS in water is limited below T_K_ due to the hydrophobicity of ODA and OA. Micelles or aggregations of ODA-TS and OA-TS begin to form at T_K_. The aggregation and adsorption parameters of liquid crystalline species can be determined from the relation between dynamic surface tension (γ; mN·m^−1^) and liquid crystalline material concentrations (lnc, mol·L^−1^). The relation between the γ and −lnc of microgels at 25 °C is illustrated in Figure 4. The relation between the γ and ageing time at different microgel concentrations were selected as representative and plotted in Figure 5.

**Figure 6:**
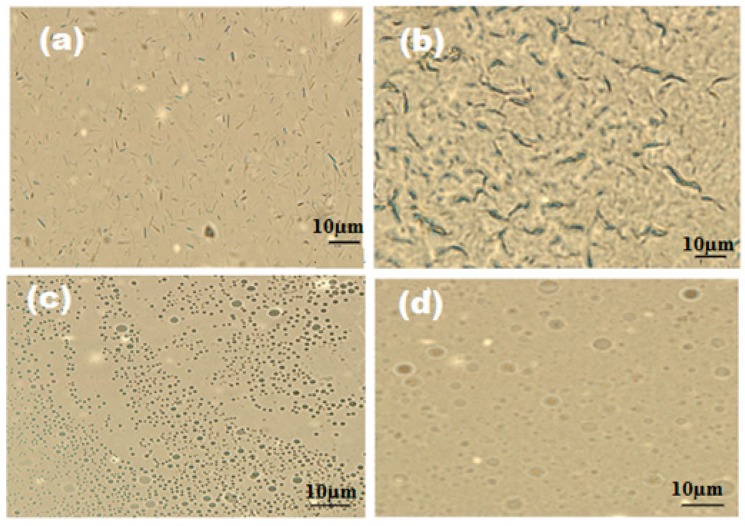
Optical microscope photographs of aqueous solutions of (**a**) ODA-TS (1 × 10^−4^ mmol/L); (**b**) ODA-TS (3 × 10^−4^ mmol/L); (**c**) OA-TS (1 × 10^−4^ mmol/L) and (**d**) OA-TS (3 × 10^−4^ mmol/L).

The optical microscope photos of OA-TS and ODA-TS at different concentrations ae presented in Figure 6. The surface tension data were listed in Table 1. The time dependent surface tension measurement relationship (Figure 6) reveals that OA-TS and ODA-TS are adsorbed very fast at the air/water interface at high concentration, reaching surface tension equilibrium in only a few seconds. The time required to equilibrate the γ measurements increases with dilution of OA-TS and ODA-TS. The micellization and adsorption of OA-TS and ODA-TS are based on the critical micelle concentrations (cmc; mol·L^−1^), which were determined from the surface balance method as presented in Figure 4. The cmc values of the prepared OA-TS and ODA-TS were measured in water at 25 °C and are listed in Table 1. The corresponding surface tension at cmc defined as γ_cmc_ was determined and also listed in Table 1. Data of surface tension measurements, Table 1 and Figure 4 and Figure 5, indicate that the prepared OA-TS and ODA-TS reduced the surface tension of water from 72.2 to 25.1 mN·m^−1^. The appearance of one adsorption curve (Figure 4) indicates the purity of OA-TS and ODA-TS. This means that the prepared OA-TS and ODA-TS behave like an amphiphile when solubilized in water and they are pure compounds. Careful inspection of the data indicated that the cmc values were reduced with the saturation of the alkyl group (ODA) which indicated that the hydrophobic moiety of ILs controls their aggregation in water. Moreover the presence of an isolated vinyl group in the alkyl hydrophobic chain (OA-TS) reduces γ_cmc_ more than ODA-TS which aggregated at a lower cmc than OA-TS. This means that the presence of a vinyl group in the structure of OA-TS increases the solubility of the surfactant in water [30]. The aggregation and micellization of OA-TS and ODA-TS can be observed by optical microscope (Figure 6). It was observed that OA-TS and ODA-TS formed aggregates above the cmc. A complete understanding of the aqueous behavior of OA-TS and ODA-TS requires knowledge of the entire self-assembly spectrum. It is well established that surfactant molecules have different micelle or aggregate shapes in the aqueous solutions (formation of micelles, liquid crystal phases, bilayers or vesicles, *etc*.). References [31,32] described the importance and existence of liquid crystalline phases. In the present work, OA-TS and ODA-TS form lyotropic liquid crystal structures at concentrations above the cmc as presented in Figure 6.

**Table 1:** Surface properties of the ILs at the air/water interface.

Compounds	Cac mol/L	γ_cac_ mN/m	(−∂ γ/∂ lnc)	Γ_max_ × 10 ^10^ mol/cm^2^	A_min_ nm^2^/molecule	−ΔG kJ·mol^−1^	
						ΔG_agg_	ΔG_ads_
ODA-TS	0.0002	31.3	8.41	3.51	0.047	20.39	21.55
OA-TS	0.0004	25.1	13.14	5.50	0.030	18.73	19.58

The optical microscopy photos (Figure 6) indicated that the ODA-TS formed nematic phase micelles that changed to rod-like structures with increasing ODA-TS concentration (Figure 6a,b). The OA-TS (Figure 6c,d) formed micelles having hexagonal and cubic forms as the OA-TS concentration increased. The presence of double bond at the center of hydrophobic part of OA-TS oriented the micelles from an elongate micelle arrangement into hexagonal arrays with increasing OA-TS concentration.

The magnitude of the hydrophobic effect and interactions can be estimated in terms of the standard free energy of transfer of the hydrophobic chain from bulk hydrocarbon to water. The aggregation processes of surfactants depend on the thermodynamic parameters of aggregation (enthalpy ΔH_mic_, entropy ΔS_mic_ and free energy ΔG_mic_). The values of ΔG_mic_ in the water can be estimated [33] as ΔG_mic_ = RT ln cmc. The ΔG_mic_ was calculated and is listed in Table 1. The low value (below zero) of ΔG_mic_ indicated that the micellization process occurred spontaneously. Moreover, it was observed that the ΔG_mic_ values are less negative with the increasing hydrophobicity of ODA-TS by saturation of the OA-TS double bond, which indicated the increment of molecule aggregation with increase of hydrophobicity. This can be explained on the basis of steric bulk of the structure that leads to steric inhibition of aggregation [32].

The adsorption of OA-TS and ODA-TS at the air/water interface is the alternative mechanism for aggregation or micellization processes which illustrate the surface activities of molecules in water and interface. The concentration of OA-TS and ODA-TS at the air/water interface was measured from surface excess concentration Г_max_. It was calculated from Equation (1):

Г_max_ = 1/RT × (−∂γ/∂ lnc)_T_ (1)

where (−∂γ/∂ lnc) _T_ is the slope of the straight lines of γ *vs.* lnc at T (constant temperature) and R gas constant (in J·mol^−1^·K^−1^). The Г_max_ values were used to calculate the minimum area A_min_ at the air/water interface. From the surface excess concentration, the area per molecule at the interface is calculated using the equation:

A_min_ = 10^16^/N Г_max_ (2)

where N is Avogadro’s number. The data of Г_max_, A_min_, and (−∂γ/∂ lnc) of OA-TS and ODA-TS were determined and are listed in Table 1. It was reported that the interaction between amphiphilic materials at the interface is very important for stabilization of particles at interfaces [34] The data listed in Table 1 indicates that the increment of Г_max_ causes an increment of adsorbed molecules at the interface between air and water, which also means a lower surface tension. The lowest value of A_min_ obtained is 0.030 nm^2^/molecule, suggesting adsorption of OA-TS which is oriented away from the liquid in a more tilted position. However, a low A_min_ data suggest complete surface coverage with the formation of flexible OA-TS chains at interface. The free energy of the microgel adsorption at air/water interface (ΔG_ad_) can be calculated from Equation (3):

ΔG_ad_ = RT ln cmc − 0.6023 π_cmc_ A_min_ (3)

where π_cmc_ = surface tension of water (72.1 mN·m^−1^) − γ_cmc_. The ΔG_ad_ values were calculated and are listed in Table 1. All ΔG_ad_ values are more negative than ΔG_mic_, indicating that the adsorption of microgels at the air/water interface is associated with a decrease in the free energy of the system. This may be attributed to the effect of steric factors on inhibition of aggregation more than its effect on the adsorption process.

### 2.3. Contact Angle and Surface Free Energy Measurements

One of the interesting criteria used to study the formation of thin films on the surface of steel is contact angle measurements. Moreover, the surface degradation of steel in 1 M HCl can be evaluated from the surface free energy values which can be determined from surface tension and contact angle measurements in the presence and absence of corrosion inhibitors [24,35]. It is well known that the surface degradation of the steel creates corrosion products and an increase in surface roughness. The surface degradation of steel can be measured in 1 M HCl as a blank and in different concentrations of OA-TS and ODA-TS molecules by measuring water contact angle values as described in the Experimental section. The surface free energy values (E) were calculated from the work of adhesion (WA) and contact angles (θ) according to the Young equations [35]:

WA = γ (1 + cosθ)
(4)

**Figure 7:**
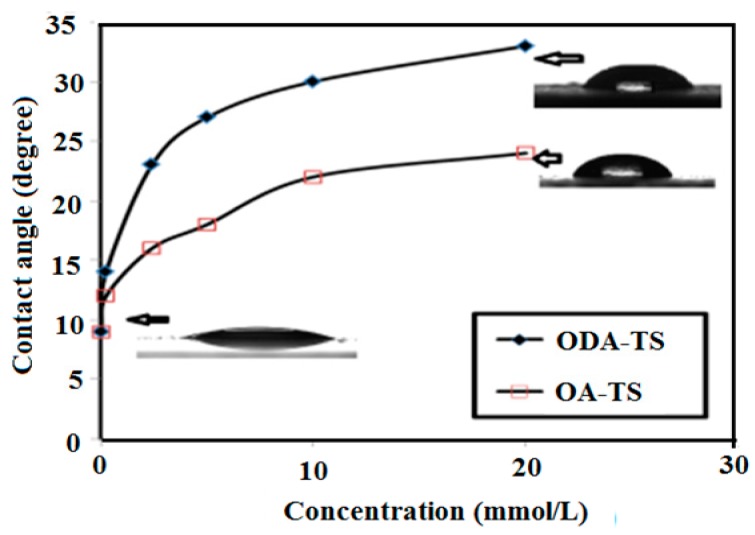
Relation between contact angle measurements and concentrations of OA-TS and ODA-TS in 1 M HCl aqueous solutions at steel surfaces.

**Figure 8:**
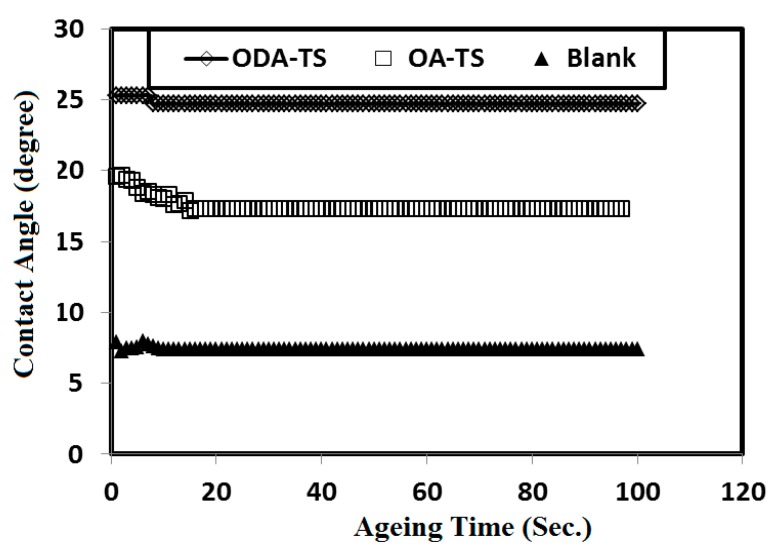
Relation between contact angle measurements and at ageing time at steel surfaces of different concentrations of OA-TS and ODA-TS in 1 M HCl aqueous solutions.

WA = 2(γ × E)^1/2^exp [−β (γ − E)^2^] (5)

where γ is the surface tension of aqueous 1 M HCl solution in the absence and presence of OA-TS and ODA-TS molecules, while the value of β is 0.0001247 ± 0.000010 (mJ/m^2^)^−2^ [24]. The relation between the contact angle values and the concentrations of OA-TS and ODA-TS is presented in Figure 7. The relation between contact angle and ageing time is illustrated in Figure 8. The data (Figure 8) indicate that ODA-TS and OA-TS in 1M HCl reached equilibrium (stable contact angle) during 5 and 20 s, respectively. It means that ODA-TS forms thin layers on the surface of steel in short time more than OA-TS. The increment of ODA-TS hydrophobicity more than OA-TS (as described in previous section) is responsible for its short time adsorption at steel surface in 1M HCl.

The surface free energy values obtained at different concentrations of OA-TS and ODA-TS are listed in Table 2. The data indicated the contact angle decreased with and the surface free energy increased after the sample exposure to HCl solution. These are responsible for a decrease in the water contact angle value and an increase in surface free energy. Moreover, it is clear from Figure 7 and Table 2 that the lowest contact angle and the highest surface free energy values were obtained for the samples immersed in HCl solution without OA-TS and ODA-TS. The data show that an increment of OA-TS and ODA-TS concentrations caused increase in surface free energy and an increase in contact angle. It can be concluded that addition of OA-TS and ODA-TS to the HCl solution caused lower steel surface degradation due to the formation of adsorbed thin film layers resulting in a lower surface roughness. Moreover, the adsorption of OA-TS and ODA-TS on the steel surface makes it more hydrophobic due to the presence of hydrophobic octadecyl and oleyl tails. These are responsible for an increase in contact angle and a decrease in surface free energy. These results can be proved from polarization test and EIS to evaluate the corrosion inhibition efficiencies of the prepared OA-TS and ODA-TS inhibitors.

**Table 2:** Surface free energy values obtained for the steel samples exposed to different concentrations of OA-TS and ODA-TS in 1 M HCl solution.

Concentrations (mmol/L)	Surface Free Energy (mJ/m^2^)	
	ODA-TS	OA-TS
0	5.1	5.1
0.272	8.9	6.5
2.84	10.6	7.6
5.70	12.4	8.5
11.36	14.2	10.8
22.73	16.1	12.2

### 2.4. Monitoring the Open Circuit Potential (E_OCP_)

**Figure 9:**
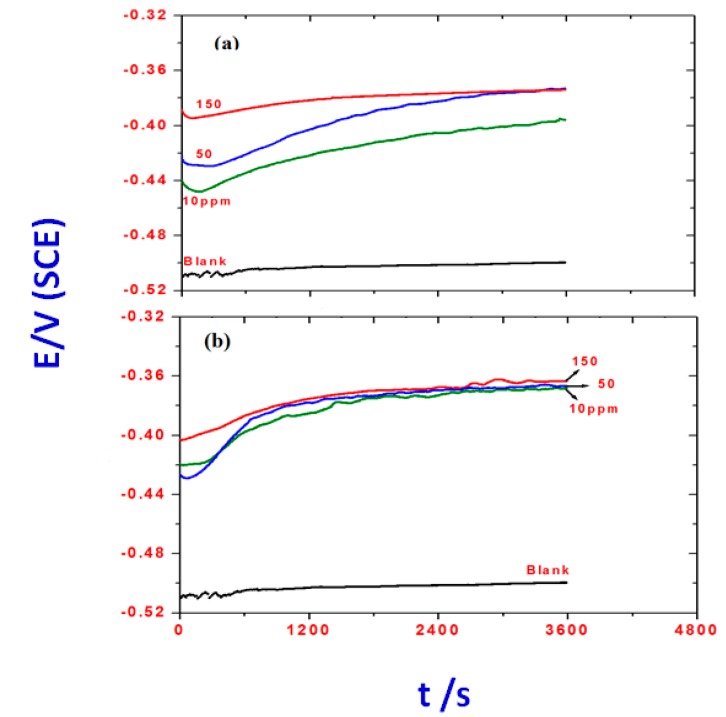
The change of open circuit potential of steel electrode as a function of immersion time in 1 M HCl solution and containing various concentrations of (**a**) OA-TS and (**b**) ODA-TS.

The evolution of the open circuit potential (E_OCP_) with time for steel in 1 M HCl solutions without and with OA-TS and ODA-TS is illustrated in Figure 9a,b, respectively.

The plots show clear modifications in the E_OCP_-time behavior due to the presence of the inhibitor. It is clear that an anodic displacement of E_OCP_ is observed with addition of OA-TS and ODA-TS. It is evident that there are potential shifts in the anodic (noble) direction in the presence of OA-TS and ODA-TS and these are more pronounced at high concentration. The inhibited solution exhibited higher positive E_OCP_ values when compared with those obtained in blank solution, indicating the formation of the protective film due to the adsorption of the inhibitor on the steel surface. The addition of OA-TS and ODA-TS to 1 M HCl solution shifts the Eocp towards more negative values during the initial stage of monitoring then it increases continuously to positive values with the increasing immersion time and eventually attains a steady state during the last stage of monitoring. The negative shift in Eocp values in the presence of inhibitor can be explained with the excessive dissolution of iron during the initial immersion. The continuous shift to noble values can be attributed to the formation of a protective and inhibitive film, which increases with increasing the inhibitor concentration and immersion time.

### 2.5. Polarization Measurements

**Figure 10:**
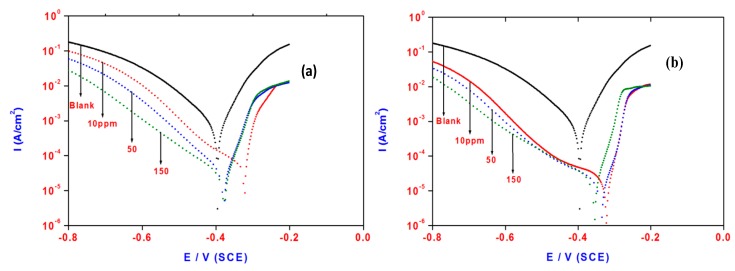
Polarization plots of steel electrode obtained in 1 M HCl solution and containing various concentrations of (**a**) OA-TS and (**b**) ODA-TS.

The electrochemical potentiodynamic polarization (EP) curves of steel in 1 M HCl solution in the presence and absence of OA-TS and ODA-TS with different inhibitor concentrations are shown in Figure 10a,b, respectively. It is clear that both anodic and cathodic currents were decreased in the presence of inhibitor and the decrease is more pronounced at higher inhibitor concentrations. The adsorption of inhibitor on steel surface decreases the cathodic hydrogen reaction and also hinders the acid attack on the steel electrode. In addition, the inhibitor also suppresses the anodic reaction and causes a reduction in the anodic current experienced during the polarization. Generally, the adsorption of OA-TS and ODA-TS compounds on active reaction sites of the steel surface inhibit both cathodic hydrogen evolution and anodic iron dissolution. Therefore, the OA-TS and ODA-TS compounds behaved as mixed-type inhibitors [36]. The inhibitory action of corrosion inhibitors can be explained on the basis of adosption of OA-TS and ODA-TS compounds on the accessible reaction surface through a geometric blocking effect. The values of electrochemical parameters, *i.e.*, corrosion potential (E_corr_), corrosion current density (I_corr_), cathodic Tafel slopes (bc) anodic Tafel slopes (ba), were calculated from the polarization curves and are listed in Table 3 and Table 4 for OA-TS and ODA-TS, respectively. It is established that the dissolution reaction is the transfer of Fe ions from the surface into the solution and the reduction of hydrogen ions is the cathodic reaction during corrosion of steel in acidic solutions. The addition of inhibitor with different concentrations altered the cathodic Tafel slopes (ba and bc) and confirms the effect of inhibitor on reduction of cathodic and anodic reactions [37]. The inhibition efficiency (IE%) is evaluated from i_corr_ values using the relationship [38,39,40]:

IE% = [1 − (I_corr2_/I_corr1_)] × 100 (6)

where I_corr1_ and I_corr2_ are corrosion current densities in the absence and presence of inhibitor, respectively. The values of IE% with different inhibitor concentrations are listed in Table 3 and Table 4 for OA-TS and ODA-TS, respectively. IE% increases with inhibitor concentrations. Increasing the inhibitor concentration resulted in an increase in the surface coverage of steel surface due to an increase in the amount adsorbed of the inhibitor on steel surface and leads to high corrosion inhibition.

**Table 3:** Inhibition efficiency values of steel electrode in 1 M HCl containing various concentrations of the OA-TS calculated by polarization and EIS methods.

Concentrations (mmol/L)		Polarization Method					EIS Method		
	ba (mV)	bc (mV)	E_corr_ (V)	I_corr_ μA/cm^2^	C.R. mm/year	IE%	Rct Ohm	Cdl (μF/cm^2^)	IE%
Blank	69	120	−0.3955	839	9.7324	____	1.80	334	____
0.023	46	202	−0.3242	73	0.2204	91.1	21.6	105	91.6
0.114	56	125	−0.3784	44	0.1392	94.7	35	103	94.8
0.341	53	156	−0.3775	31	0.1160	96.3	54	101	96.6

**Table 4:** Inhibition efficiency values of steel electrode in 1 M HCl containing various concentrations of the ODA-TS calculated by polarization and EIS methods.

Concentrations (mmol/L)			Polarization Method				EIS Method		
	ba (mV)	bc (mV)	E_corr_ (V)	I_corr_ μA/cm^2^	C.R. mm/year	IE%	Rct Ohm	Cdl (μF/cm^2^)	IE%
Blank	69	120	−0.3955	839	9.7324	____	1.80	334	____
0.023	40	206	−0.3235	19	0.8468	97.7	65.7	95	97.2
0.114	42	153	−0.3369	12	0.5104	98.5	80.7	93	97.7
0.341	48	116	−0.3606	10	0.3596	98.8	90.3	91	98.0

### 2.6. Electrochemical Impedance Spectroscopy (EIS)

**Figure 11:**
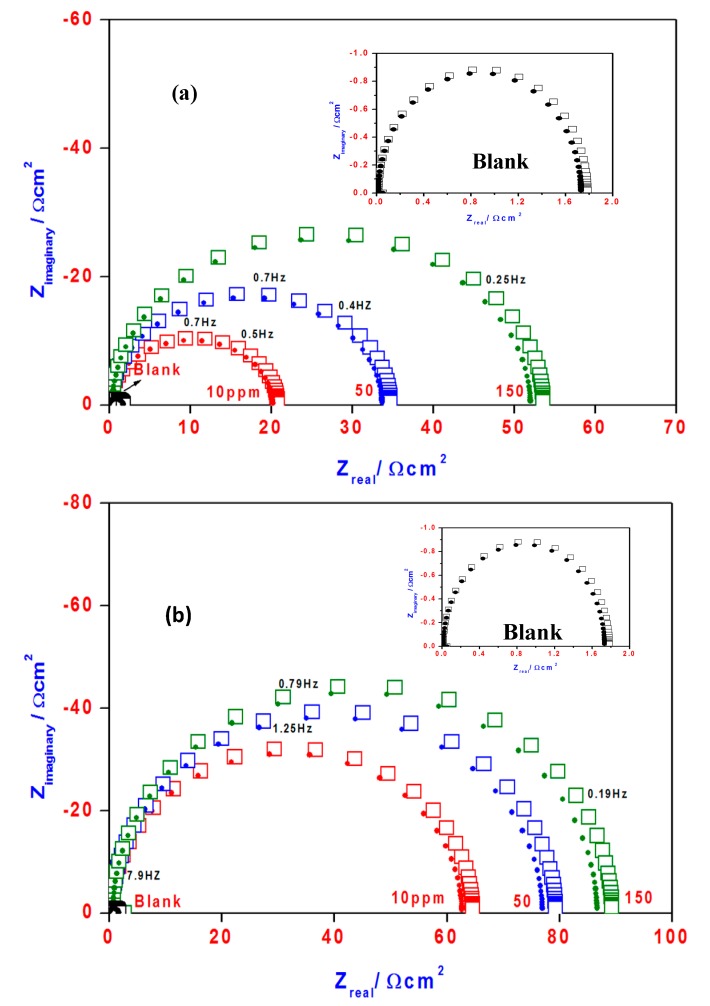
Nyqusit plots of steel electrode obtained in 1 M HCl solution and containing various concentrations of: (**a**) ODA-TS and (**b**) OA-TS (square and circle symbols indicate experimental and fit data, respectively).

**Figure 12:**
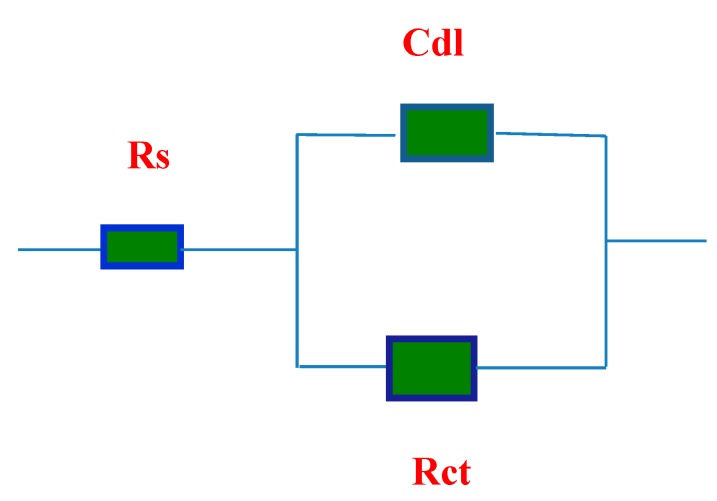
Equivalent circuit employed for fitting EIS data.

The effect of the OA-TS and ODA-TS concentrations on the impedance behavior of steel in 1 M HCl solution is presented as Nyquist plots in Figure 11a,b, respectively. The impedance diagrams show a capacitive loop, whose size increased with increasing inhibitor concentration. The capacitive loop was related to charge transfer in the corrosion process [41]. The electrical equivalent circuit employed for fitting the data composed of solution resistance (Rs), charge transfer resistance (Rct) and double layer capacitance (Cdl) as shown in Figure 12. The impedance parameters as (Rct) and (Cdl) are calculated and are listed in Table 3 and Table 4 for OA-TS and ODA-TS, respectively. The increase in the thickness of the electrical double layer and/or decrease in local dielectric constant resulted from the decrease in the double capacitance and increase of charge transfer resistance indicating that the inhibitor acts by adsorption at the steel/solution interface [42]. The gradual replacement of water molecules by adsorption of the inhibitor molecules on the steel surface causes a change in the values of Rct and Cdl and decreases the extent of acidic dissolution of the steel [43]. The data presented in Table 3 and Table 4 indicated that that the inhibitor had strongly adsorbed to the surface of steel. The inhibition efficiency (%IE) is calculated from Rct using the following relation:

IE% = [1 − (Rct_1_/Rct_2_)] × 100 (7)

where Rct_1_ and Rct_2_ are the charge transfer resistances in the absence and in the presence of the inhibitors, respectively. The percentage inhibition efficiency (IE%) was calculated from Equation (2) and listed in Table 3 and Table 4 for OA-TS and ODA-TS, respectively. The maximum inhibition efficiency (98%) was achieved at an inhibitor concentration of 150 ppm. A strong adsorption of inhibitor on steel surface indicates a more surface coverage by the inhibitor and accompanies by an increase in the charge-transfer resistance (Rct) values [44]. The inhibition efficiencies calculated from EIS are in good agreement with those obtained from potentiodynamic polarization.

### 2.7. SEM and EDX Measurements

**Figure 13:**
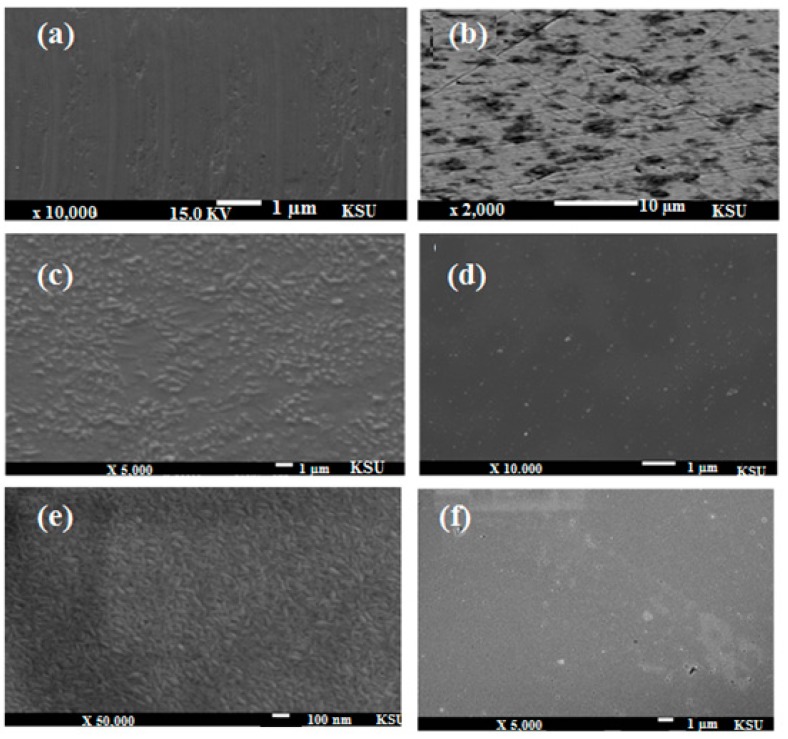
SEM micrographs of (**a**) polished steel bare; (**b**) steel bare immersed in 1 M HCl; (**c**) steel coated with 100 ppm of OA-TS; (**d**) steel coated with 100 ppm of OA-TS after immersion in 1 M HCl; (**e**) steel coated with 100 ppm of ODA-TS before immersion in 1 M HCl and (**f**) steel coated with 100 ppm of ODA-TS after immersion in 1 M HCl.

To confirm the formation of the films on the steel surface, the SEM technique was used to characterize the iron surface. Figure 13a–d,f presents the morphology of the polished bare steel in 1 M HCl aqueous solution and in 1 M HCl containing OA-TS and ODA-TS, respectively. Figure 13a shows the steel surface after polishing, which appeared as a smooth surface with some scratches. Inspection of Figure 13b reveals that the steel after a 5 h immersion in uninhibited 1 M HCl shows aggressive attack damage as a great deal of deep cavities were found. Figure 13c, present the images of the iron sheet covered by OA-TS and ODA-TS films after immersion in 100 ppm of aqueous solutions.

**Figure 14:**
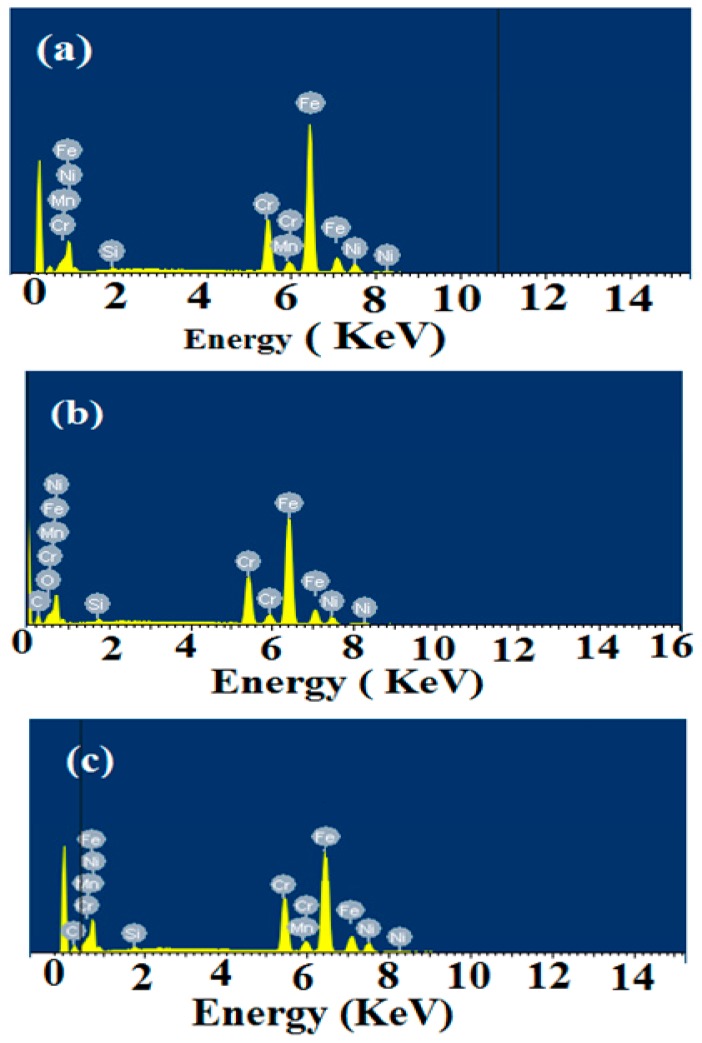
EDX analyses of (**a**) steel; (**b**) steel coated with 100 ppm of ODA-TS and (**c**) steel coated with 100 ppm of OA-TS after immersion in 1 M HCl aqueous solutions.

However, under the same corrosion circumstances, the surface of film-modified steel sheet (Figure 13d,f) was a smooth and cleaner surface and only a few small pits are observed on the steel surface. Inspection of the morphologies implied that the presence of the OA-TS and ODA-TS films can efficiently protect the steel from corrosion. Moreover, the formation of self-assembled films at the surface of steel increases the interaction between OA-TS and ODA-TS and the steel surface, resulting in a decrease in the contact between the steel and the aggressive medium. These results are in good agreement with the results obtained by EIS and potentiodynamic polarization measurements. This behavior suggested that both compounds mitigate steel corrosion rates with complete inhibition all over the steel surface. EDX is an interesting technique used to analyze the protective film formed on a polished steel surface. It is used to confirm the composition of the formed protective film. The instrument does not give the corresponding peaks for N, and O, but confirmed the corresponding peaks for metals. In this respect, the strength of the Fe peak in the absence and presence of the inhibitor gives an idea about the thickness of the protective film. The EDX spectra of carbon steel samples were presented in Figure 14a. The data indicate that the strength of the iron band decreased when steel was immersed in the OA-TS and ODA-TS inhibitors (Figure 14c). The EDX data confirm the formation of a strongly adherent protective film of self-assembled OA-TS and ODA-TS adsorbed on the steel surface, which inhibits the corrosion of steel [45].

### 2.8. Mechanism of Corrosion Inhibition

The presence of OA-TS and ODA-TS in 1 M HCl blocked the local cathodes and anodes by adsorbing on the steel surface. It is established that the adsorption of the inhibitor on the steel surface displaces water molecules and other species, forming an adsorbed and inhibitive film at the steel surface. The higher the surface coverage of steel the slower the corrosion process of steel as evident from the extremely large reduction in anodic and cathodic current density. The adsorption of the inhibitor on the steel surface in acidic chloride solution is mainly due to a geometric blocking effect. Therefore, the protection efficiency (IE) equals the coverage of the steel surface with the adsorbed inhibitor species.

**Figure 15:**
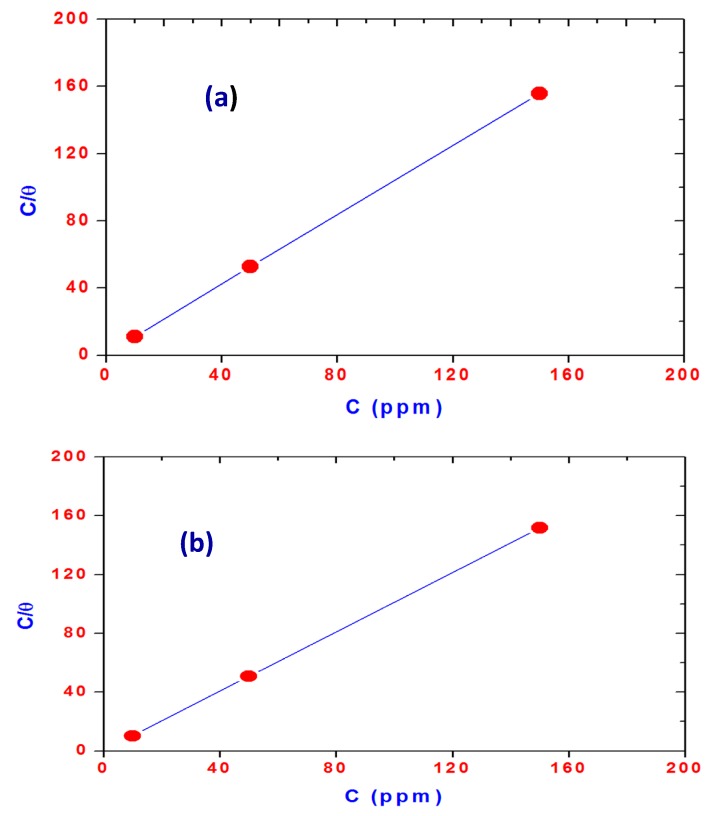
Langmuir adsorption plot of steel in 1 M HCl solution containing different concentrations of (**a**) OA-TS and (**b**) ODA-TS.

The experimental results were fitted to series of adsorption isotherms, *i.e.*, Langmuir, Temkin, Bockris-Swinkels, Flory-Huggins and Frumkin. The adsorption behavior of both inhibitors obeys Langmuir adsorption isotherm, which is given by [46]:

C_(inh)_/θ = 1/K_ads_ + C_(inh)_ (8)

where C_(inh)_ is inhibitor concentration and Kads is the equilibrium constant for the adsorption process. A plot of C/θ *vs.* C (Figure 15) gives a straight line with an average correlation coefficient of 0.9999 and 1 for OA-TS and ODA-TS as shown in Figure 14a,b, respectively. The near unity slope suggests that the adsorptions of both inhibitors on steel obey a Langmuir adsorption isotherm. From the values of the adsorption constant, K_ads_, the standard free energy of adsorption (ΔG°ads) for the inhibitor is determined using the following equation [47,48]:

ΔG° = −RT ln (55.5 K_ads_) (9)

ΔG° was calculated for OA-TS and ODA-TS and found to be −38.456 kJ·mol^−1^ and −38.474 kJ·mol^−1^, respectively. The high value of ΔG° provides evidence of strong interactions between the inhibitor and the steel surface [49,50,51,52]. It was established that when the standard free energy of adsorption is around −20 kJ·mol^−1^ or lower a process of physical adsorption may occur predominantly through electrostatic interactions between the charged inhibitor and the charged exposed surface [53,54,55,56,57]. A chemical adsorption takes place [43,44,45,46,47] when the standard free energy of adsorption (ΔG°ads) values are around −40 kJ·mol^−1^ or higher. The negative values of the standard free energy of adsorption (ΔG°ads) indicate a spontaneous adsorption of the inhibitor on the steel surface by displacement of water molecules from the steel surface by the inhibitor and formation of a protective film [57]. The oxidation reactions of iron are significantly inhibited as a result of spontaneous adsorption of the inhibitor molecules on the iron surface and form a protective layer [48,58,59]. The adsorption of the inhibitor at cathodic sites of steel reduces H_2_ gas evolution [49,50,53]. In addition, the high molecular weight and large size of the inhibitor molecules extremely affect the protection efficiency of the inhibitors [60]. The ODA-TS and OA-TS molecules are ionized in aqueous solution to alkylammmonium cations and tosylate ions, as illustrated in the Scheme 1. These molecules can be adsorbed physically on the steel surfaces through an electrostatic interaction mechanism between ODA and OA ammonium cations and the steel surface that possesses negative charges produced from reaction with HCl to produce (FeCl^−^) in the anodic reaction [61]. The adsorption of alkylammonium cations at the steel surface forms an adsorbed monomolecular or bimolecular layer by forming a complex on the steel surface. The adsorbed layers protect the steel surfaces from Cl^−^ attack to prevent the steel oxidation reaction at the anode. Moreover, the protonated alkyl ammonium cations of ODA and OA adsorb at cathodic sites to compete with H^+^ to prevent hydrogen evolution. Moreover, the tosylate anion can interacts with H^+^ produced by the HCl to prevent the hydrogen evolution. The presence of a double bond in the structure of OA can form an interaction between the π electrons and the unoccupied d-orbital of iron to produce a new center of adsorption action, which cannot be obtained for ODA cations.

## 3. Experimental Section

### 3.1. Materials

Oleylamine (OA), *p-*toluenesulfonic acid monohydrate (TSA; 99.5%), and octadecylamine (ODA; 97%) were purchased from Aldrich Chemicals Co. (St Louis, MO, USA) and used with no further purification. Hydrochloric acid (37 wt %) was obtained from Merck (Darmstadt, Germany). Tests were performed on steel specimens with weight percentage composition as follows: 0.14% C, 0.57% Mn, 0.21% P, 0.15% S, 0.37% Si, 0.06% V, 0.03% Ni, 0.03% Cr and Fe is the balance. The aggressive acid environment was 1 M HCl. The surface of the working electrode was prepared by grinding with different grades of emery papers, then rinsing with distilled water and ultrasonic degreasing in ethanol and eventual drying at room temperature.

### 3.2. Synthesis Procedure

Equimolar amounts of ODA or OA and *p*-toluenesulfonic acid were mixed in a flask equipped with a magnetic stirrer, thermometer and reflux condenser. The reaction mixture was heated for 24 h under nitrogen atmosphere at temperature 145 °C. Finally, the solid octadecylammonium tosylate (ODA-TS) and oleyl ammonium tosylate (OA-TS) products were obtained after cooling to room temperature. The melting temperatures of OA-TS and ODA-TS salts are 56 and 85 °C, respectively. The products were purified by heating at 45 °C under reduced pressure (at 10 mm·Hg) by using vacuum rotary evaporator for 24 h. The yields of products ODA-TS and OA-TS are 99.5 wt % and 99 wt %. The C, H, N and S % determined for ODA-TS are 67.92%, 10.63%, 3.17% and 7.24%, respectively. The C, H, N and S % detected from elemental analysis for OA-TS are 68.24%, 10.21%, 3.19% and 7.26%, respectively.

### 3.3. Characterization

^1^H- and ^13^C-NMR spectra of the prepared polymers were recorded on a 400 MHz Avance DRX-400 spectrometer (Bruker, Baden-Württemberg, Germany). A drop shape analyzer model DSA-100 (Krüss GmbH, Hamburg, Germany) was used to perform the surface tension measurements of IL solutions and measure the contact angles. Krafft Temperature T_K_ determined from the relation between conductivity and temperature as reported before [62]. The conductance was determined using an AB30 conductivity meter (Fisher Scientific, Waltham, MA, USA). A scanning electron microscope (SEM, Model JSM-5400, JEOL, Tokyo, Japan) was used to determine the microstructure of IL films formed on the steel surfaces. The chemical analyses of the steel surfaces were determined using an energy dispersive X-ray analysis (EDX) accessory which was attached to the SEM-JEOL 5400. The analyses were carried out after immersion of steel working electrodes (0.5 cm^2^) in the inhibitor solutions for 6 h and drying with nitrogen at room temperature. An optical microscope (BX51, Olympus, Tokyo, Japan) equipped with a digital camera (XD200, Flovel Co., Ltd., Tokyo, Japan) was used to determine the micelle morphology.

### 3.4. Electrochemical Measurements

Electrochemical measurements were performed on a Solartron 1470E system (Potentiostat/Galvanostat) with a Solartron 1455A frequency response analyzer to perform all polarization and EIS measurements (Solartron, Farnborough, UK). Polarization curves were recorded at a constant sweep rate of 1 mV/s. EIS was in the frequency range of 10 kHz to 10 mHz. A three-electrode cell assembly was employed to perform electrochemical experiments. Platinum sheet was used as a counter electrode and saturated calomel electrode (SCE) as the reference electrode. The electrochemical experiments data were collected and analyzed by using CorrView, Corr-Ware software. The analysis of impedance data and selection of an equivalent circuits to fit the experimental results were conducted using the Zplot and ZView software.

## 4. Conclusions

The main conclusion of the present study may be summarized in the following points:

(1) OA-TS and ODA-TS can act as an effective corrosion inhibitor for steel in 1 M hydrochloric acid.
(2) OA-TS and ODA-TS act as a mixed type inhibitor retarding the anodic and cathodic reactions.
(3) The electrochemical results indicated that the protection efficiency of the inhibitor is highly dependent upon concentration and attains a maximum value (98%) at an inhibitor concentration of 150 ppm.
(4) EIS data indicated that the charge transfer resistances increase with the addition of inhibitor and the corrosion process is mainly controlled by charge transfer reactions.
(5) SEM and EDX techniques revealed the formation of protective films on the steel surface.

## References

[1] Oguzie E., Li Y., Wang F. (2007). Corrosion inhibition and adsorption behavior of methionine on mild steel in sulfuric acid and synergistic effect of iodide ion. J. Colloid Interface Sci..

[2] Seter M., Thomson M.J., Stoimenovski J., MacFarlane D.R., Forsyth M. (2012). Dual active ionic liquids and organic salts for inhibition of microbially influenced corrosion. Chem. Commun..

[3] Gopi D., Govindaraju K., Kavitha L. (2010). Investigation of triazole derived Schiff bases as corrosion inhibitors for mild steel in hydrochloric acid medium. J. Appl. Electrochem..

[4] Gopi D., Govindaraju K., Collins Prakash A., Manivannan V., Kavitha L. (2009). Inhibition of mild steel corrosion in groundwater by pyrrole and thienylcarbonyl benzotriazoles. J. Appl. Electrochem..

[5] Popoola L.T., Grema A.S., Latinwo G.K., Gutti B., Balogun A.S. (2013). Corrosion problems during oil and gas production and its mitigation. Int. J. Ind. Chem..

[6] Bentiss F., Lagrenee M., Traisnel M., Mernari B., Elattari H. (1999). 3,5-Bis(*n*-Hydroxyphenyl)-4-amino-1,2,4-triazoles and 3,5-bis(*n*-aminophenyl)-4-amino-1,2,4-triazoles: A new class of corrosion inhibitors for mild steel in 1 M HCl medium. J. Appl. Electrochem..

[7] Subramania A., Sundaram N.K., Priya R.S., Saminathan K., Muralidharan V., Vasudevan T. (2004). Aldimines—Effective corrosion inhibitors for mild steel in hydrochloric acid solution. J. Appl. Electrochem..

[8] Malik M.A., Hashim M.A., Nabi F., Al-Thabaiti S.A., Khan Z. (2011). Anti-corrosion ability of surfactants: A review. Int. J. Electrochem. Sci..

[9] El-Maksoud S.A. (2008). The effect of organic compounds on the electrochemical behaviour of steel in acidic media. A review. Int. J. Electrochem. Sci..

[10] Atta A.M., El-Azabawy O.E., Ismail H., Hegazy M. (2011). Novel dispersed magnetite core–shell nanogel polymers as corrosion inhibitors for carbon steel in acidic medium. Corros. Sci..

[11] El-Mahdy G.A., Atta A.M., Al-Lohedan H.A. (2014). Synthesis and characterizations of Fe_3_O_4_ nanogel composite for enhancement of the corrosion resistance of steel in HCl solutions. J. Taiwan Inst. Chem. Eng..

[12] Behzadnasab M., Mirabedini S., Esfandeh M. (2013). Corrosion protection of steel by epoxy nanocomposite coatings containing various combinations of clay and nanoparticulate zirconia. Corros. Sci..

[13] Gergely A., Pfeifer E., Bertoti I., Torok T., Kalman E. (2011). Corrosion protection of cold-rolled steel by zinc-rich epoxy paint coatings loaded with nano-size alumina supported polypyrrole. Corros. Sci..

[14] Saremi M., Yeganeh M. (2014). Application of mesoporous silica nanocontainers as smart host of corrosion inhibitor in polypyrrole coatings. Corros. Sci..

[15] Sababi M., Pan J., Augustsson P.E., Sundell P.E., Claesson P.M. (2014). Influence of polyaniline and ceria nanoparticle additives on corrosion protection of a UV-cure coating on carbon steel. Corros. Sci..

[16] Doner A., Solmaz R., Ozcan M., Karda G. (2011). Experimental and theoretical studies of thiazoles as corrosion inhibitors for mild steel in sulphuric acid solution. Corros. Sci..

[17] Likhanova N.V., Dom nguez-Aguilar M.A., Olivares-Xometl O., Nava-Entzana N., Arce E., Dorantes H. (2010). The effect of ionic liquids with imidazolium and pyridinium cations on the corrosion inhibition of mild steel in acidic environment. Corros. Sci..

[18] Zhang Q., Hua Y. (2009). Corrosion inhibition of mild steel by alkylimidazolium ionic liquids in hydrochloric acid. Electrochim. Acta.

[19] Lin P.C., Sun I.W., Chang J.K., Su C.J., Lin J.C. (2011). Corrosion characteristics of nickel, copper, and stainless steel in a Lewis neutral chloroaluminate ionic liquid. Corros. Sci..

[20] Tüken T., Demir F., Kicir N., Sigircik G., Erbil M. (2012). Inhibition effect of 1-ethyl-3-methylimidazolium dicyanamide against steel corrosion. Corros. Sci..

[21] Wang Y.C., Lee T.C., Lin J.Y., Chang J.K., Tseng C.M. (2014). Corrosion properties of metals in dicyanamide-based ionic liquids. Corros. Sci..

[22] Gu T., Chen Z., Jiang X., Zhou L., Liao Y., Duan M., Wang H., Pu Q. (2015). Synthesis and inhibition of *N*-alkyl-2-(4-hydroxybut-2-ynyl) pyridinium bromide for mild steel in acid solution: Box–Behnken design optimization and mechanism probe. Corros. Sci..

[23] Perissi I., Bardi U., Caporali S., Lavacchi A. (2006). High temperature corrosion properties of ionic liquids. Corros. Sci..

[24] Kowsari E., Payami M., Amini R., Ramezanzadeh B., Javanbakht M. (2014). Task-specific ionic liquid as a new green inhibitor of mild steel corrosion. Appl. Surf. Sci..

[25] Huang P., Latham J.-A., MacFarlane D.R., Howlett P.C., Forsyth M. (2013). A review of ionic liquid surface film formation on Mg and its alloys for improved corrosion performance. Electrochim. Acta.

[26] Reynolds J.L., Erdner K.R., Jones P.B. (2002). Photoreduction of benzophenones by amines in room-temperature ionic liquids. Org. Lett..

[27] Zhao G., Jiang T., Gao H., Han B., Huang J., Sun D. (2004). Mannich reaction using acidic ionic liquids as catalysts and solvents. Green Chem..

[28] Luo J., Hu J., Saak W., Beckhaus R., Wittstock G., Vankelecom I.F.J., Agert C., Conrad O. (2011). Protic ionic liquid and ionic melts prepared from methanesulfonic acid and 1*H*-1,2,4-triazole as high temperature PEMFC electrolytes. J. Mater. Chem..

[29] Flores C.A., Flores E.A., Hernández E., Castro L.V., García A., Alvarez F., Vázquez F.S. (2014). Anion and cation effects of ionic liquids and ammonium salts evaluated as dehydrating agents for super-heavy crude oil: Experimental and theoretical points of view. J. Mol. Liq..

[30] Rosen M.J., Kunjappu J.T. (2012). Surfactants and Interfacial Phenomena.

[31] Laughlin R.G. (1994). The Aqueous Phase Behavior of Surfactants.

[32] Chandrasekhar S. (1992). Liquid Crystals.

[33] Binks B., Fletcher P., Kotsev S., Thompson R. (1997). Adsorption and aggregation of semifluorinated alkanes in binary and ternary mixtures with hydrocarbon and fluorocarbon solvents. Langmuir.

[34] Arditty S., Schmitt V., Giermanska-Kahn J., Leal-Calderon F. (2004). Materials based on solid-stabilized emulsions. J. Colloid Interface Sci..

[35] Li D., Neumann A. (1990). A reformulation of the equation of state for interfacial tensions. J. Colloid Interface Sci..

[36] Wang X., Yang H., Wang F. (2010). A cationic gemini-surfactant as effective inhibitor for mild steel in HCl solutions. Corros. Sci..

[37] Bentiss F., Traisnel M., Lagrenee M. (2000). The substituted 1,3,4-oxadiazoles: A new class of corrosion inhibitors of mild steel in acidic media. Corros. Sci..

[38] Qu Q., Hao Z., Li L., Bai W., Liu Y., Ding Z. (2009). Synthesis and evaluation of Tris-hydroxymethyl-(2-hydroxybenzylidenamino)-methane as a corrosion inhibitor for cold rolled steel in hydrochloric acid. Corros. Sci..

[39] Bentiss F., Lebrini M., Lagrenée M. (2005). Thermodynamic characterization of metal dissolution and inhibitor adsorption processes in mild steel/2,5-bis(*n*-thienyl)-1,3,4-thiadiazoles/hydrochloric acid system. Corros. Sci..

[40] Qu Q., Jiang S., Bai W., Li L. (2007). Effect of ethylenediamine tetraacetic acid disodium on the corrosion of cold rolled steel in the presence of benzotriazole in hydrochloric acid. Electrochim. Acta.

[41] Deyab M., El-Rehim S.A. (2012). On surfactant–polymer association and its effect on the corrosion behavior of carbon steel in cyclohexane propionic acid. Corros. Sci..

[42] McCafferty E., Hackerman N. (1972). Double layer capacitance of iron and corrosion inhibition with polymethylene diamines. J. Electrochem. Soc..

[43] Muralidharan S., Phani K., Pitchumani S., Ravichandran S., Iyer S. (1995). Polyamino benzoquinone polymers: A new class of corrosion inhibitors for mild steel. J. Electrochem. Soc..

[44] Fekry A., Mohamed R.R. (2010). Acetyl thiourea chitosan as an eco-friendly inhibitor for mild steel in sulphuric acid medium. Electrochim. Acta.

[45] Amin M.A. (2006). Weight loss, polarization, electrochemical impedance spectroscopy, SEM and EDX studies of the corrosion inhibition of copper in aerated NaCl solutions. J. Appl. Electrochem..

[46] Lebrini M., Bentiss F., Vezin H., Lagrenee M. (2006). The inhibition of mild steel corrosion in acidic solutions by 2,5-bis(4-pyridyl)-1,3,4-thiadiazole: Structure-activity correlation. Corros. Sci..

[47] Goulart C.M., Esteves-Souza A., Martinez-Huitle C.A., Rodrigues C.J.F., Maciel M.A.M., Echevarria A. (2013). Experimental and theoretical evaluation of semicarbazones and thiosemicarbazones as organic corrosion inhibitors. Corros. Sci..

[48] Kele H., Kele M., Dehri I., Serinda O. (2008). The inhibitive effect of 6-amino-m-cresol and its Schiff base on the corrosion of mild steel in 0.5 M HCI medium. Mater. Chem. Phys..

[49] Bayol E., Gürten A., Dursun M., Kayakirilmaz K. (2008). Adsorption behavior and inhibition corrosion effect of sodium carboxymethyl cellulose on mild steel in acidic medium. Acta Phys. Chim. Sin..

[50] Avci G. (2008). Inhibitor effect of *N*,*N*-methylenediacrylamide on corrosion behavior of mild steel in 0.5 M HCl. Mater. Chem. Phys..

[51] Lebrini M., Robert F., Vezin H., Roos C. (2010). Electrochemical and quantum chemical studies of some indole derivatives as corrosion inhibitors for C38 steel in molar hydrochloric acid. Corros. Sci..

[52] Bahrami M., Hosseini S., Pilvar P. (2010). Experimental and theoretical investigation of organic compounds as inhibitors for mild steel corrosion in sulfuric acid medium. Corros. Sci..

[53] Abiola O., Oforka N. (2004). Adsorption of (4-amino-2-methyl-5-pyrimidinyl methylthio) acetic acid on mild steel from hydrochloric acid solution (HCl)—Part 1. Mater. Chem. Phys..

[54] Mert B.D., Mert M.E., Karda G., Yazici B. (2011). Experimental and theoretical investigation of 3-amino-1,2,4-triazole-5-thiol as a corrosion inhibitor for carbon steel in HCl medium. Corros. Sci..

[55] Wang X., Yang H., Wang F. (2011). An investigation of benzimidazole derivative as corrosion inhibitor for mild steel in different concentration HCl solutions. Corros. Sci..

[56] Negm N., Elkholy Y., Zahran M., Tawfik S. (2010). Corrosion inhibition efficiency and surface activity of benzothiazol-3-ium cationic Schiff base derivatives in hydrochloric acid. Corros. Sci..

[57] Tebbji K., Faska N., Tounsi A., Oudda H., Benkaddour M., Hammouti B. (2007). The effect of some lactones as inhibitors for the corrosion of mild steel in 1M hydrochloric acid. Mater. Chem. Phys..

[58] Solmaz R., Karda G., Yazici B., Erbil M. (2008). Adsorption and corrosion inhibitive properties of 2-amino-5-mercapto-1,3,4-thiadiazole on mild steel in hydrochloric acid media. Colloids Surf. A Physicochem. Eng. Asp..

[59] Solmaz R., Karda G., Culha M., Yazc B., Erbil M. (2008). Investigation of adsorption and inhibitive effect of 2-mercaptothiazoline on corrosion of mild steel in hydrochloric acid media. Electrochim. Acta.

[60] Behpour M., Ghoreishi S., Salavati-Niasari M., Ebrahimi B. (2008). Evaluating two new synthesized S–N Schiff bases on the corrosion of copper in 15% hydrochloric acid. Mater. Chem. Phys..

[61] Li S.L., Wang Y.G., Chen S.H., Yu R., Lei S.B., Ma H.Y., Liu X.D. (1999). Some aspects of quantum chemical calculations for the study of Schiff base corrosion inhibitors on copper in NaCl solutions. Corros. Sci..

[62] Vautier-Giongo C., Bales B.L. (2003). Estimate of the Ionization Degree of Ionic Micelles Based on Krafft Temperature Measurements. J. Phys. Chem. B.

